# Potential Biomarkers in Experimental Animal Models for Traumatic Brain Injury

**DOI:** 10.3390/jcm12123923

**Published:** 2023-06-08

**Authors:** Uma Maheswari Deshetty, Palsamy Periyasamy

**Affiliations:** Department of Pharmacology and Experimental Neuroscience, University of Nebraska Medical Center, Omaha, NE 68198, USA; udeshetty@unmc.edu

**Keywords:** traumatic brain injury, biomarkers, animal models

## Abstract

Traumatic brain injury (TBI) is a complex and multifaceted disorder that has become a significant public health concern worldwide due to its contribution to mortality and morbidity. This condition encompasses a spectrum of injuries, including axonal damage, contusions, edema, and hemorrhage. Unfortunately, specific effective therapeutic interventions to improve patient outcomes following TBI are currently lacking. Various experimental animal models have been developed to mimic TBI and evaluate potential therapeutic agents to address this issue. These models are designed to recapitulate different biomarkers and mechanisms involved in TBI. However, due to the heterogeneous nature of clinical TBI, no single experimental animal model can effectively mimic all aspects of human TBI. Accurate emulation of clinical TBI mechanisms is also tricky due to ethical considerations. Therefore, the continued study of TBI mechanisms and biomarkers, of the duration and severity of brain injury, treatment strategies, and animal model optimization is necessary. This review focuses on the pathophysiology of TBI, available experimental TBI animal models, and the range of biomarkers and detection methods for TBI. Overall, this review highlights the need for further research to improve patient outcomes and reduce the global burden of TBI.

## 1. Introduction

Traumatic brain injury (TBI) is a substantial global public health concern and a leading cause of death [1]. The worldwide incidence of TBI is approximately 50 million cases, with a gradual increase in prevalence (8.4% between 1990 to 2016), and 80% of TBI cases are reported from developing countries [2]. In the United States alone, nearly 1.7 million individuals suffer from TBI annually, and 224,000 hospitalizations due to TBI are reported [3]. TBI is caused by various factors, such as direct hits, falls, vehicle accidents, rapid acceleration or deceleration, penetrating foreign objects, episodes of violence (blasts/explosions), and contact sports, which result in alterations in brain functions or brain damage [4,5,6,7]. Falls are the leading cause of TBI among individuals below 14 years and over 65 years, whereas vehicle accident related TBI is common among individuals aged 15–24 years and over 65 years [8].

There are two classifications of TBI: penetrating brain injury (PBI), characterized by a skull fracture, and closed head injury (CHI), which is devoid of a skull fracture. PBI is a more severe form of TBI caused by skull fractures from sharp objects or gunshot wounds. CHI is more commonly observed in individuals involved in sports or military services and caused by direct impact on the face, head, neck, or other parts of the body and by projectiles or bullets affecting the helmet [9,10,11,12]. Different types of CHI include contusion, diffuse axonal injury, concussion, and intracranial hematomas such as subdural hematoma, epidural hematoma, intra-parenchymal hemorrhage, or subarachnoid hemorrhage [13]. TBI can be classified as mild, moderate, and severe based on its severity. Mild TBI is associated with less cognitive impairment that may resolve within 3 months in patients. Moderate or severe TBI is associated with long-term cognitive impairments that may remain unresolved for months or a lifetime of the patient [14,15,16].

Understanding the severity of TBI and guiding appropriate management strategies relies heavily on the clinical definition and classification of this condition. The Glasgow Coma Scale (GCS), widely recognized and extensively employed in clinical practice, assesses TBI severity by evaluating three primary components: eye-opening response, verbal response, and motor response [17]. Each component receives a score ranging from 1 to 6, with higher scores signifying better functioning. The GCS scores enable the classification of TBI into three levels: mild, moderate, and severe. Mild TBI, typically indicated by a GCS score of 13–15, involves a brief loss of consciousness, post-traumatic amnesia lasting less than 24 h, and normal imaging results. Moderate TBI, corresponding to a GCS score of 9–12, encompasses a loss of consciousness lasting 30 min to 24 h, post-traumatic amnesia persisting for 1–7 days, and imaging abnormalities like contusions or hemorrhages. Severe TBI, associated with a GCS score of 3–8, entails a prolonged loss of consciousness exceeding 24 h, post-traumatic amnesia lasting more than 7 days, and significant imaging abnormalities such as contusions, hemorrhages, or diffuse axonal injury [17]. The GCS serves as a dependable tool for healthcare professionals to promptly assess TBI severity, appropriately triage patients, establish treatment plans, and make prognostic considerations.

TBI is characterized by immediate impairments (primary injury or mechanical damage) and sustained impairments (secondary injury), which lead to cell death followed by axonal damage, tissue necrosis, and eventually functional impairments [18,19]. Animal models of TBI help analyze the pathology and physiology of injury progression and generate efficient injury management strategies [19]. Various animal models, such as rodents, cats, pigs, dogs, and non-human primates, were developed to explore the underlying pathophysiology of TBI [20]. However, the heterogeneous nature of the primary injury (diffuse, focal, or multifocal) followed by varied secondary responses (cellular and biochemical responses) makes the diagnosis, prognosis, and management of TBI challenging [21]. Currently, another limitation in the management of TBI is the lack of specific molecular biomarkers that could be helpful in accurately evaluating the patient’s injury and further tracking the recovery and treatment of the patient [22,23,24]. Biomarkers are measurable indicators in the body that can provide valuable information about the injury’s severity, progression, and prognosis. They can include various substances, such as proteins or molecules, that are released or altered in response to brain injury. On the other hand, imaging techniques, such as computed tomography (CT) scans and magnetic resonance imaging (MRI), enable the visualization of structural and functional changes in the brain, aiding in the identification and characterization of TBI-related abnormalities. Both biomarkers and imaging offer valuable insights into the complex nature of TBI, allowing for early detection, accurate assessment, and personalized treatment strategies. This review summarizes various biomarkers of TBI in experimental animal models that could serve as potential diagnostic tools for the proficient diagnosis and management of TBI.

## 2. Physiology of TBI

The physiology of TBI is complex, and it involves many pathophysiological events that cause structural disruptions and functional deficits through both primary and secondary injury mechanisms.

### 2.1. Primary Injury

The primary injury in TBI refers to the immediate physical damage to the brain tissue upon impact with an external force. Primary injuries include cerebral contusions, concussions, penetrating wounds, and lacerations. These injuries cause mechanical damage to the brain tissue, resulting in necrotic cell death and the tearing of blood vessels (hemorrhage), glial cells, neurons, and axons [25,26,27]. These initial injuries are mechanical but irreversible, and no therapeutic strategies exist to reverse the damage. Safety devices such as helmets or seat belts are the only effective way to prevent primary injury [28,29,30].

### 2.2. Secondary Injury

Secondary injury in TBI is a complex process that occurs after the initial impact, initiated by tissue deformation from the primary injury. It encompasses various processes that can exacerbate neurological damage, such as cerebral edema, intracranial hypertension, ischemia, excitotoxicity, oxidative stress, inflammation, and apoptosis. This cascade of events ultimately leads to brain damage and functional deficits [31]. The depolarization of neurons after the primary injury triggers the release of excitatory neurotransmitters such as glutamate and aspartate [32,33]. These neurotransmitters bind to glutamate receptors and cause a substantial influx of calcium ions into the cells, which activates various enzymes such as calcium-dependent proteases, endonucleases, and phospholipases that degrade proteins, nucleic acids, and lipids. This calcium influx also causes mitochondrial calcium sequestration, leading to disturbances in calcium homeostasis, free radicals’ formation, energy deficits, and initiation of apoptosis [34,35,36]. The formation of increased reactive oxygen and reactive nitrogen species after TBI leads to the oxidation of proteins, nucleic acids, and lipids, causing further damage to the brain tissue [37].

TBI also causes the upregulation of several transcription factors, inflammatory mediators, and neuroprotective genes while down-regulating neurotransmitter receptors and their release mechanisms [38]. The upregulation of chemokines and cytokines also causes the disruption of the blood-brain barrier (BBB), brain edema, increased intracranial pressure, impaired cerebral flow, ischemia, hypoxia, increased lactate levels, and energy deficits leading to brain damage such as cellular damage/death, axonal injury, brain atrophy, and demyelination followed by functional deficits [39]. However, research over the past decade has shown that the adult brain can undergo structural and functional reorganization after injury, which can be helpful for spontaneous functional recovery. Interventions that target mechanisms involved in secondary injury and neuroplasticity modulation have also been shown to enhance functional recovery in TBI animal models [30].

### 2.3. Types of TBI

Mild TBI is the most common type of TBI, accounting for approximately 80–90% of all cases, and is typically caused by sports participation, everyday life, and military service [40]. Usually, the patient experiencing mild TBI remains awake but suffers from brain function disruption, such as brief loss of consciousness, headache, disorientation, confusion, and memory loss. A pathophysiological cascade of heterogeneous events is initiated due to mild TBI, which involves a consortium of metabolic, inflammatory, axonal, and neuronal changes [41,42,43,44]. The first 24 h or first week of injury usually witness substantial adverse effects on cognition and balance among patients with mild TBI. Athletes typically recover from these impairments within the first month, while civilians may take up to three months [1,6]. However, cognitive deficits could persist for several hours, three months, or up to one year after the initial event [45,46,47,48,49]. In most cases, patients may develop post-concussion syndrome, which comprises long-term psychogenic and organic symptoms, such as behavioral and cognitive changes [50]. It is worth noting that repeated mild TBI may have long-term implications, including Alzheimer’s disease, Parkinson’s disease, and chronic traumatic encephalopathy [1,51,52,53,54,55,56,57,58,59]. Moderate to severe TBI, often caused by vehicle accidents, can lead to long-term cognitive impairments with potential lifetime consequences and permanent disability [1]. These injuries involve a longer duration of loss of consciousness, exceeding 30 min up to 24 h for moderate cases and extending beyond 24 h for severe cases. Post-traumatic amnesia is frequently observed, with moderate TBI resulting in amnesia lasting 1 to 7 days and severe TBI leading to amnesia persisting for more than 7 days. Imaging techniques like CT scans or MRIs often detect notable abnormalities such as contusions, hemorrhages, or diffuse axonal injury. Functional outcomes encompass motor deficits, cognitive impairments, emotional instability, mood disorders, and personality changes. Rehabilitation and comprehensive medical care play a crucial role in optimizing recovery and promoting the best possible outcomes for individuals with moderate to severe TBI. Understanding the different types of TBI and their potential long-term effects is critical in developing effective prevention and treatment strategies for this condition.

## 3. Animal Models of TBI

### 3.1. Animals

Due to the complex nature of logistic and ethical issues with clinical studies involving humans, most TBI research is conducted in animal models [1]. Additionally, vast pathophysiological heterogeneity is observed among patients with TBI, which may be due to the nature, severity, and position of the primary injury, as well as other pre-existing factors such as age, gender, health, medication, drug or alcohol abuse, and genetics [60].

#### 3.1.1. Rodents

Rodent models have become the go-to solution for TBI research because they offer several advantages over clinical studies involving human subjects. First, they allow researchers to conduct experiments without exposing humans to the risk of potential harm or discomfort. This is particularly important for TBI research, where subjects may have sustained severe injuries that experimental procedures could further exacerbate. Rodent models also offer a level of control that is difficult to achieve in clinical studies. Researchers can manipulate various factors, such as the nature, severity, and position of the injury, and pre-existing factors, like age, gender, and genetics, to create a controlled environment for experimentation. This level of control allows researchers to isolate specific variables and study their effects on the injury and recovery process.

Additionally, rodent models offer a way to obtain statistically significant data by including large numbers of animals per group, which can help detect actual differences between groups. This is particularly advantageous for TBI research, where the heterogeneity observed among patients with TBI makes it difficult to draw meaningful conclusions from smaller sample sizes. Also, rodent models are economical due to the low cost of procuring, handling, and housing animals for experimental purposes [1]. Despite the advantages of rodent models, however, there are still limitations and ethical considerations to be addressed. Rodent models do not always accurately reflect the complexity of human TBI, and the findings from animal studies may not always translate to human patients. Also, the use of animal models in experimentation requires ethical considerations and issues to be taken into account to ensure that animals are treated humanely and that the research is conducted with the highest standards of care.

#### 3.1.2. Non-Rodents

The use of non-rodent animal models for TBI research has gained traction due to the limitations of rodent models [61]. While rodents are the most commonly used TBI models, consistent concerns have been over their translational potential to humans. This is primarily due to a lissencephalic cortex in rodents, which differs from the gyrencephalic brains of higher species such as primates [62,63]. Moreover, earlier studies using large animal species have reported consistent variations in intracranial pressure and cerebral perfusion pressure after TBI, which are not as pronounced in rodents. Therefore, non-rodent animal models have been increasingly used to study TBI, especially mild ones [50].

Several non-rodent species, such as dogs, cats, rabbits, pigs, and non-human primates, have been used in TBI research. These animal models have been instrumental in investigating proof-of-concept models for TBI research, detecting underlying genotype-phenotype relationships, pathophysiological mechanisms, and long-term outcomes [20]. However, their experimentation involves ethical considerations and issues, sophisticated facilities for conducting surgery and post-operative care, the requirement of experienced veterinarian personnel, and huge costs [1]. While rodent models remain useful in TBI research, the growing body of evidence supporting the usage of non-rodent animal models highlights the need for a more comprehensive understanding of TBI pathophysiology across species. Employing diverse animal models in TBI research can better represent the complexities involved in TBI, facilitating the development of effective therapeutic strategies.

### 3.2. Experimental Animal Models

Recreating TBI in a reproducible manner has proven challenging due to the involvement of heterogeneous and multi-planar physical forces, including rotational, shearing, concussive, and ischemic intracerebral injuries [64,65,66]. In recent years, experimental animal models have focused on implementing a single aspect of these harmful multifactorial mechanisms, followed by studying the subsequent pattern of brain injury. Earlier, researchers have demonstrated several animal models for TBI studies, which have proven immensely useful in understanding the underlying pathophysiology of TBI [64,67,68].

Experimental TBI animal models can be broadly divided into two injury categories: penetrating (open-head models) and non-penetrating injury (closed-head models). Once distinguished into the above two categories, the models were designed to induce either focal (confined to one particular location on the brain) or diffuse injury (spread across more than one location on the brain) with specific modifications. Focal injury TBI animal models are further classified as weight drop models, fluid percussion injury (FPI), and controlled cortical impact (CCI). The models possess the varied ability to induce mild, moderate, or severe injury [69].

#### 3.2.1. Closed-Head Injury Models

Closed-head injury TBI animal models involve imparting an impulse or force through an intact skull, with or without skin incision and exposure of the outer table, to induce brain injury. This category includes impact models (weight-drop model) and non-impact models (piston-driven model). Generally, impact models cause focal injury, whereas non-impact models apply an inertial force to the head, leading to angular brain momentum and causing diffuse injury [66].

##### Weight-Drop Model

This model involves dropping a projectile (brass load) of specified features via a plexiglass tube from a specified height onto the head of the animal [70]. The weights are generally varied and propelled with the help of gravity or an electromagnetic or pneumatic actuator to apply a mechanical load directly to the skull of the animal [71,72]. Researchers have observed considerable variation in the weight/drop height usage of the projectile and other factors such as whether the mice were anesthetized during impact, whether the head was immobilized, whether surgery was performed, direct or indirect skull impact, impact location, projectile shape, and material [70]. Another limitation of this model is that it does not accurately replicate the complex and diverse nature of human TBI. It is a simplified model that only focuses on a single type of physical force applied to the head, while in reality, TBI is often caused by a combination of forces, including rotational, shearing, concussive, and ischemic injuries. Additionally, this model only focuses on acute injury and does not account for the long-term effects and potential chronic conditions that can arise from TBI. Lastly, the model has limitations regarding its reproducibility and consistency, as the variability in impact parameters and animal responses can make it difficult to compare and replicate results between studies.

##### Piston-Driven Model

This model involves causing injury at a specific velocity, depth, or impact force by zeroing the piston on the surface of the scalp or skull. Researchers use various piston devices, such as pneumatically driven, compressed nitrogen, or electromagnetic pistons, for this model. This model further categorizes based on the placement of the animal and the location of the injury. In the CHIMERA model, the researchers place the animal on its back inside the device and impart the injury from below, allowing the animal to flex its head after the impact [73]. The Hit and Run model induce injury by hanging the animal from a string by the incisors and imparting a piston strike to the side of the head, allowing the animal to move freely after the impact [74]. Other models induce impact using a lateral angle, allowing animals to lie on a flat surface and move laterally after the impact [75,76,77]. This model also has some limitations that should be considered when interpreting results obtained using this model. One of the main limitations is that it only produces a focal injury rather than the diffuse injury seen in human TBI. Additionally, the severity of the injury can vary depending on the location of the impact and the depth of the piston, which can make it difficult to compare results across studies. Another limitation is that the injury is typically induced in anesthetized animals, which may not accurately reflect the physiological responses in awake animals or humans. Finally, the use of animals in this model raises ethical concerns and may limit its translation to human clinical trials. Despite these limitations, the piston driven TBI model remains valid for studying some aspects of TBI pathology and evaluating potential treatments.

#### 3.2.2. Open-Head Injury Models

In open-head injury models of TBI in animals, researchers perform a craniotomy to expose the dura mater and apply impulses directly to it. These models can be divided into four specific types: fluid percussion injury (FPI), controlled cortical impact (CCI), blast injury model, and weight-drop-impact acceleration injury [71,78,79,80,81]. Due to the impact of in situ extradural impulses, these models lack significant head movement and can induce moderate to severe grade focal cortical contusion (direct cortical impact) or diffuse brain or axonal injury, i.e., fluid percussion [66,82].

##### Fluid Percussion Injury Models

The fluid percussion injury model induces injury to the intact dura mater by generating a fluid pressure pulse using a pendulum striking the piston of a fluid reservoir. Craniotomy is performed either centrally (around the midline) or laterally (over the parietal bone), between bregma and lambda, to expose the dura mater. The percussion briefly causes brain tissue displacement and deformation, and the strength of the pressure pulse determines the severity of the injury [83,84]. This model also replicates human TBI without skull fracture and exhibits pathophysiological hallmarks of clinical TBI, such as intracranial hemorrhage, swelling of the brain, and progressive gray matter disruption [85,86]. Based on the craniotomy position away from the sagittal suture, this model can be classified into midline (centered on the sagittal suture), parasagittal (lateral to the midline, <3.5 mm), and lateral models (lateral to the midline, > 3.5 mm) [84,87,88,89]. The midline fluid percussion injury model was initially developed in cats and rabbits and subsequently used in rats, and then modifications were made to generate the lateral fluid percussion injury model in rodents. The pathophysiology and pharmacology of TBI have been studied using the fluid percussion injury model in dogs, cats, sheep, rabbits, pigs, rats, and mice. The lateral fluid percussion injury model is one of the most commonly used animal models and causes both focal cortical contusion and diffuse subcortical neuronal injury (such as hippocampus and thalamus). Within minutes of the impact, loss of neurons is observed, and 12 h up to 7 days post-injury, the injury does not expand into other brain regions. Over the weeks, beneath the injury site, the contused cortex enlarges to become a cavity lined with glia which continues to expand until one-year post-injury, mainly due to ongoing cell death. Over months, a progressive degenerative cascade of events occurs in vulnerable brain regions, such as the thalamus, ipsilateral hippocampus, striatum, medial septum, and amygdala. The lateral fluid percussion injury model produces cognitive and neurobehavioral deficits such as memory loss and movement impairments commonly observed in TBI patients and persist for more than a year [84,87,88,89]. While the model has been valuable in advancing our understanding of TBI and testing potential treatments, it has some drawbacks. These include a lack of consistency, limited translational value, potential confounding variables, and ethical concerns. Overall, while the fluid percussion injury model has been helpful in TBI research, it is crucial to be aware of its potential limitations and use it with other models to gain a more comprehensive understanding of TBI.

##### Controlled Cortical Impact (CCI) Model

The Controlled Cortical Impact (CCI) model is a rigid percussion model that shares similar concepts with the impact accelerator, weight drop, and fluid percussion models [67]. In this model, researchers anesthetize animals and securely affix their heads to the experimental apparatus using stereotactic pinning to prevent cranial motion. They then execute a limited circular craniotomy, usually between bregma and lambda, and use an electromagnetically, pneumatically, or electromechanically driven rigid piston to apply a direct extradural impulse [79,90,91]. The CCI model mimics acute subdural hematoma, cortical tissue loss, thalamic and hippocampal degeneration, concussion, axonal injury, BBB impairments, and coma [68,79,92,93,94,95]. Researchers have developed the CCI model in rats, mice, ferrets, pigs, and monkeys [79,92,93,96,97]. The advantage of the CCI model is that researchers can control the depth and velocity of the impulse, affecting the severity of brain damage. Another advantage is the lack of risk of a rebound injury [90]. It has been observed in rats and mice that functional deficits, such as cognitive deficits quantified using the Morris water maze test, were significantly related to both the velocity and depth of deformation of the impact [98,99,100]. The researchers reported that cognitive deficits persisted for up to a year and could be associated with a progressive reduction in cerebral blood flow and brain atrophy in post-CCI [101,102,103].

Furthermore, CCI was found to cause impaired emotional behavior in mice, as measured using the elevated plus maze, prepulse inhibition of acoustic startle, and forced swim test [103]. Researchers have also reported that the swine CCI model develops reproducible injury with pathophysiological features similar to human TBI, thus making it a viable option to translate in vivo data to clinical practice [96,104]. However, the CCI model of TBI has several limitations. One major limitation is that it is a focal injury model, meaning that it only mimics the effects of a single impact at a specific site in the brain and does not accurately reflect the diffuse nature of most TBIs, which involve multiple brain areas. Also, the CCI model typically produces severe injuries not representative of the full spectrum of TBI severity seen in human patients. Another limitation is that the model relies on a single impact, whereas many human TBIs result from multiple impacts. Finally, using anesthesia and stereotactic pinning in the CCI model may introduce confounding variables that could affect the interpretation of results.

##### Blast Injury Model

Several military personnel exposed to blasts without external injuries have been diagnosed with TBI [105,106]. Researchers have developed many animals’ blast TBI models, mainly in rodents and pigs, to evaluate the effects of the primary blast wave on the CNS [80,107,108,109,110,111,112]. In this model, a compression-driven shock tube is used to simulate blast effects, and researchers assess the consequences of blast exposure on neuropathological, physiological, and neurobehavioral aspects [111]. Researchers evaluated the effect of a Kevlar thoracic protective vest encasing the thorax and abdominal parts on mortality in rats and the frequency of TBI among the survivors. The Kevlar vest significantly reduced air blast mortality and attenuated axonal fiber degeneration, demonstrating that shock tube-generated blast induces TBI in rats through systemic effects, such as hypoxemia and hypotension [113]. Another group of researchers generated a blast induced TBI model in rats to mimic absolute blast mild TBI observed in military conflicts. Non-impact blast injury showed remarkable pathophysiologies, such as severe diffuse hyperemia, cerebral brain edema, and delayed vasospasm typically observed in human and animal blast brain injury [114,115]. In rat models with body shielding, the diffuse axonal injury was among the most prominent features during the initial 2 weeks after blast exposure [116]. Severe explosive blast exposure to the head alone caused significant neurological dysfunction. Interestingly, rats exposed to low-level blast enhanced intracranial pressure levels, causing cognitive deficits [117,118].

**Figure 1:**
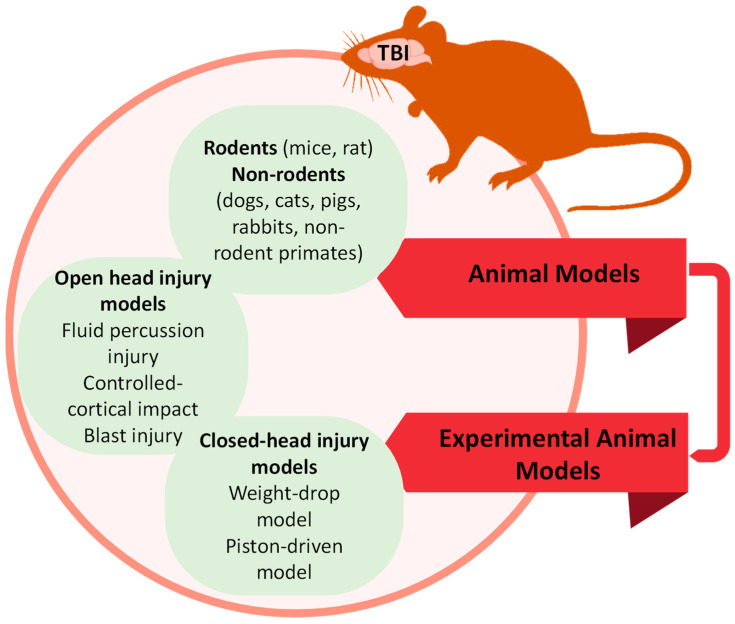
Schematic shows various animal models used in TBI research.

Although functional deficits caused by blast exposure represent the major health problem in modern warfare, most animal blast models focus on tissue damage rather than functional deficits [61,113,116]. A recent study demonstrated that brain injury due to mild blast caused prolonged behavioral and motor abnormalities in mice, such as impairments in spatial memory, social recognition, motor coordination, and torso shielding attenuated axonal injury and behavioral deficits [119]. The position of the animal along the shock tube affects how much force it experiences and can change the type and severity of the injury, as well as the likelihood of death [120]. Like any other animal model, the blast injury model has certain limitations. One of the main limitations is the difficulty in replicating the complex nature of human blast injuries in animals. While the primary blast wave effect can be simulated, the secondary and tertiary effects, such as blunt trauma, penetrating injuries, and burns, are challenging to mimic accurately. Another limitation is the potential species-specific differences in response to blast injury. Animal models may not always fully represent human biology, and responses to blast injury may differ between species. Moreover, blast injury models may focus more on tissue damage than on the functional deficits that are the major health problems faced by blast-exposed individuals. Therefore, further research is necessary to improve animal models for blast injury and better understand the functional deficits resulting from blast exposure (Figure 1).

### 3.3. Unconventional TBI Models

Recent studies have shown that unconventional simulation techniques can serve as a viable alternative to animal models for investigating TBI [121]. These techniques encompass various approaches to replicate different aspects of the injury, including dynamic mechanical forces like linear and angular acceleration. Blast injury models, mainly used in rodents and recently adapted for pigs, utilize controlled explosions or compressed air to generate blast shockwaves and induce brain damage. Direct force models can be categorized into non-impact and impact head acceleration models, with non-impact models involving rapid linear or rotational acceleration in non-human primates and pigs. Direct impact models involve either penetrating injury with direct brain deformation or non-penetrating injury with impact acceleration. Penetrating injury is simulated using constrained head models like fluid percussion and CCI, while unconstrained head models such as weight-drop and bolt guns are used for non-penetrating injury simulation [121]. However, it is important to consider the limitations of experimentally inflicted injury models, which may involve non-clinically relevant procedures and additional damage unrelated to TBI. One study used finite element analysis to simulate TBI biomechanics and determine injury thresholds, demonstrating the feasibility and accuracy of simulation models in predicting injury patterns and severity. These studies highlight the potential of simulation models in TBI research, providing controlled experiments, precise measurement of injury parameters, and detailed analysis of mechanical forces [122]. Simulation techniques offer an ethical and cost-effective alternative to animal models, although further validation and refinement are still needed. Nonetheless, they hold significant promise for advancing our understanding of TBI and improving preventive measures and treatment strategies [122].

## 4. Biomarkers of TBI

### 4.1. Behavioral Biomarkers

The motor functions and behavior typically coordinate motor function through complex neural network systems, which originate in the cortex and terminate in skeletal muscles. The sensorimotor cortex, association cortex, subcortical nuclei, cerebellum, and brainstem communicate to signal the spinal cord to mediate movement [123]. Impairments caused by brain injury in these pathways can lead to motor deficits, such as difficulties with movement initiation, execution, and coordination. In animal models, researchers commonly use sensorimotor function tests to evaluate the outcome of brain injury, as TBI can cause damage to complex motor pathways and sensorimotor integration [123].

To evaluate the outcome of brain injury in animal models, researchers commonly use sensorimotor function tests, as TBI can cause damage to complex motor pathways and sensorimotor integration [123]. The rotarod, cylinder, grip strength, staircase, and skilled forelimb reach tests are among the most commonly used sensorimotor function tests [123]. For example, the rotarod test evaluates motor coordination and balance by measuring the time a rodent can maintain balance on a rotating cylinder. The cylinder test assesses forelimb asymmetry and detects sensorimotor deficits in the acute and chronic stages of TBI. The skilled forelimb reach test is another well-known test that evaluates fine motor function, often impaired in the early stages of TBI. Researchers frequently use the neurological severity score, which includes behavior and motor functions, in rodents with closed-head injury models [124,125]. In rodents with unilateral brain injury models, they use the modified neurological severity score to detect neurological functional deficits [126].

Although TBI seldom leads to cognitive dysfunction in humans, the severity of cognitive dysfunction usually depends on the severity of the injury [127]. However, researchers have described cognitive deficits in lateral and midline fluid percussion injury, CCI, impact acceleration, and blast animal models [32,124,128,129,130,131]. The most commonly used rodents’ cognitive tests are response tests, Morris water maze tests, novel object recognition tests, and memory tasks [123,132]. For instance, the Morris water maze test assesses spatial learning and memory, which are often impaired after TBI. Researchers have also developed a few other complex behavioral tests in TBI research studies to mimic the complex psychological and personality disturbances seen in TBI patients. Anxiety-like tests comprise emotional and exploratory activity, elevated plus maze, and open field tests [98,133,134]. Although depression is a common clinical problem after TBI, it has not been thoroughly studied in animal models. However, a few studies report using the forced swimming test to evaluate depression-like behavior [134,135]. These tests evaluate an animal’s ability to swim and climb to escape a stressful situation and are thought to be analogous to the human condition of learned helplessness. Additionally, it is essential to note that there may be missing details or limitations associated with these animal models and tests in some cases. For instance, the degree to which these models mimic the complexity and variability of human TBI may be limited, and care must be taken when extrapolating findings to human populations. Abnormal results obtained from these tests can indicate memory dysfunctions, which may be associated with various physiological and psychological disorders of the nervous system. Further studies utilizing these tests have the potential to provide valuable insights and therapeutic approaches for addressing these diseases [132].

### 4.2. Biochemical Biomarkers

TBI is typically diagnosed by assessing the severity of primary cerebral lesions and secondary brain damage caused by several biochemical and molecular mechanisms, such as reactive oxygen species (ROS), lipid peroxidation, upregulated glutamate release, and neuronal inflammation [136]. However, the current standard clinical diagnosis of TBI involves evaluating using the GCS, which categorizes TBI based on a scoring system ranging from mild (score of 13–15), moderate (score of 9–12), to severe (score of ≤8) [137]. The GCS assesses the patient’s eye, motor, and verbal responses, with a score of 0 indicating no response (i.e., acute coma or death) and the highest score indicating the normal ability to respond to tasks (completely awake or alert). Despite its widespread use, the GCS has several disadvantages, including scoring inconsistencies, scale changes, and emotional discrepancies or interpretations by healthcare professionals [138]. Similarly, the Glasgow Outcome Scale (GOS) measures neurological or functional outcomes and is commonly scored as 1 (death), 2 (persistent vegetative state), 3 (severe disability), 4 (moderate disability), and 5 (good recovery) [139]. However, GCS and GOS are not specific to TBI and can also be used to assess the severity of brain-related injuries such as Alzheimer’s disease and stroke [140,141].

Researchers have explored several specific and non-specific biochemical markers to improve TBI diagnosis and outcome prediction. Specific biomarkers for TBI include neuron-specific enolase (NSE), myelin basic protein (MBP), S100 calcium-binding protein B (S100B), ubiquitin carboxyl-terminal hydrolase L1 (UCH-L1), and glial fibrillary acidic protein (GFAP). Non-specific biomarkers include those for damage (autophagy or apoptosis-specific), inflammation (proinflammatory and anti-inflammatory cytokines), metabolic changes (glucose, C-reactive protein), and degeneration (nerve growth factor, vascular endothelial growth factor, tau protein) [69]. Additionally, it is necessary to analyze specific biomarkers that identify differences in long-term cognitive deficits or changes. Elevated levels of NSE and low soluble neuron cell adhesion molecule (sNCAM) may help predict the risk of functioning and attention deficits following TBI in pediatric patients [142]. Researchers have also observed decreased levels of brain-derived neurotrophic factor (BDNF), crucial for the maintenance and regeneration of neurons, in patients who have suffered from TBI. These lower levels of BDNF are believed to be associated with disruptions in head computed tomography (CT) and a prognosis of a 6-month recovery [143]. Combining GCS and serum biomarker concentrations has also improved outcome prediction, increasing sensitivity and specificity [144]. Hence, a combination of biomarkers is required for TBI diagnosis and outcome prediction.

Various molecules are released after TBI, and some are now recognized as novel biomarkers specific to TBI. These biomarkers have the potential to identify, quantify, and predict outcomes of TBI. Among these biomarkers, alpha II-spectrin breakdown products (SBDP) is an axonal injury biomarker [145,146]. Tau protein is another axonal injury biomarker, with a plasma phospho-tau and phospho-tau:total-tau ratio for acute and chronic TBI biomarkers [147]. Heart fatty acid binding protein (H-FABP) is a biomarker for CT-positive mild TBI and severe TBI [148]. Neurofilament heavy (NF-H) is a traumatic axonal injury biomarker [149]. MicroRNAs are specific microRNAs that could be used as indicators for the diagnosis, severity, and prognosis of mild-to-moderate TBI [150]. Aldolase C, brain lipid binding protein, astrocytic phosphoprotein PEA15, and glutamine synthetase are astroglial TBI-defined biomarkers for acute and late-phase severe TBI [151]. Substance P, soluble CD40 ligand, tissue inhibitor for metalloproteinase-1, malondialdehyde, and cytokeratin-18 are TBI mortality biomarkers [152]. Progesterone, GFAP, and IL6 could aid in prognosticating outcomes in patients with acute severe TBI through serial monitoring [153]. Mitochondrial enzymes C1, C4, and PDH could be used as mitochondrial-targeted therapies and aid in prognostication [154]. Cystatin D is an inflammatory TBI biomarker that could aid in pre-hospital care to detect TBI [155]. UCHL-1 is a neuronal marker for severe TBI [156]. Finally, marinobufagenin (MBG) is a neuroinflammation biomarker but is nonspecific to the brain [157]. Identifying and quantifying TBI-specific biomarkers can significantly enhance the diagnosis, assessment of severity, and prediction of prognosis for TBI. The biomarkers mentioned above are just a few examples of the growing number of potential TBI biomarkers being studied. As research in this area continues, it is hoped that new and more specific biomarkers will be identified, leading to better TBI care and outcomes for patients.

### 4.3. MicroRNAs

In recent years, researchers have shown a growing interest in using microRNAs as a potential biomarker to identify and predict the outcomes of TBI, as well as to develop therapeutic or interventional strategies post-TBI. MicroRNAs have been shown to regulate various physiological and pathological functions at the cellular level, including development, catabolism or anabolism, and apoptosis. Experimental studies have evaluated specific microRNAs, such as miR-21 and miR-23b, as biomarkers and therapeutic targets for mild to moderate TBI in animals. Recent studies have identified additional serum and CSF microRNAs in humans as possible indicators of TBI diagnosis, severity, and prognosis. These include miR-21, miR-425-p, miR-191, miR-93, and miR-499 in serum, and miR-328, miR-451, miR-362-3p, and miR-486a in CSF [150].

Furthermore, a group of researchers has delved into the map of brain-derived extracellular vesicle (EV) miRNA and utilized machine learning to determine a set of biomarkers that could classify specific injury states. This panel of eight miRNAs, comprising miR-150-5p, miR-488-3p, miR-669c-5p, miR-9-5p, miR-22-5p, miR-6236, miR-351-3p, and miR-219a.2-3p, was identified for TBI-induced mice versus sham mice. In addition, four miRNAs, namely miR-203a-3p, miR-203b-5p, miR-185-5p, and miR-206, were found to be potential biomarkers for differentiating between patients with TBI and healthy controls [158]. Overall, microRNAs show promise as potential biomarkers for identifying, quantifying, and predicting outcomes of TBI. Further research is needed to determine their clinical utility and potential for use in developing targeted therapies for TBI.

### 4.4. Neuroimaging Techniques

Neuroimaging techniques are commonly used for diagnosing TBI, with non-invasive imaging techniques such as magnetic resonance imaging (MRI) and CT being the most frequently used methods [69]. MRI and CT can help detect physical changes in the brain structure, such as axonal and white matter injury. Recently, advanced MRI techniques have been developed to precisely detect the pathophysiological changes in the injured brain, which may contribute to the collective and long-term effects of repeated mild TBIs [159,160,161].

TBI causes physical changes to the brain structure, such as axonal and white matter injury. The corpus callosum, a brain structure that connects the two hemispheres, is commonly injured in TBI. Its location allows the evaluation of its structural integrity and function using imaging. Fractional anisotropy is used to measure corpus callosum white matter integrity. The corpus callosum functionality can be evaluated by interhemispheric transfer time, which measures the time for information to cross cerebral hemispheres [69]. It has been demonstrated that callosal function is related to disrupted white matter integrity in pediatric TBI [162]. Another recent study comparing cortical thickness in different brain sub-regions between post-mild TBI patients and controls with orthopedic injuries highlighted the complexity of using neuroimaging techniques to predict TBI outcomes. The study showed cortical thickness varied by brain sub-regions and was not a reliable predictor of TBI outcomes [163]. In addition, neuroimaging has been explored in predicting post-TBI behavioral impairments [164]. Various metabolites detected during neuroimaging have been evaluated for their use as TBI biomarkers, including creatine, N-acetyl aspartate, lactate, choline, and myoinositol [165,166,167,168]. However, neuroimaging techniques can be risky and expensive and provide minimal information for targeting patient treatment or evaluating patient prognosis [69]. Although CT scans are becoming increasingly common in head injury cases, there is a risk of unnecessary exposure to radiation, which is especially dangerous for pediatric patients [169,170].

**Figure 2:**
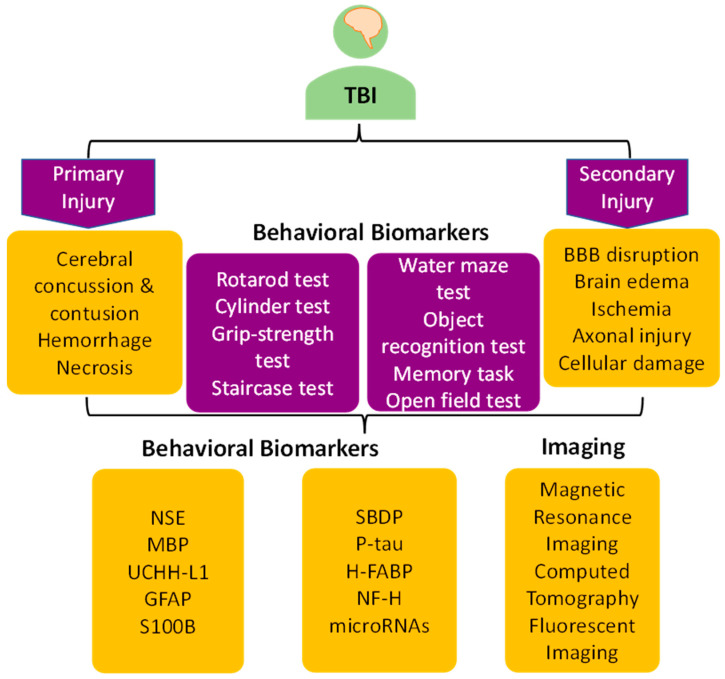
Schematics show various biomarkers used in TBI research.

Recently, fluorescence imaging has gained increasing attention in biological imaging due to its high spatial and temporal resolution, sensitivity, simplicity, remarkable contrast, and non-invasiveness [171,172,173]. Compared to conventional fluorescence imaging in the visible and NIR-I spectral range (400–900 nm), NIR-II fluorescence imaging significantly reduces tissue scattering, autofluorescence, and light absorption, allowing deeper tissue penetration, higher spatial resolution, and dynamic in vivo imaging of the brain without craniotomy. Additionally, the appearance of organic fluorophores with large photon absorption cross sections and high fluorescence quantum efficiency has significantly enhanced the development of two-photon or three-photon imaging for diagnosing TBI. With the continuous development of fluorescence imaging technology, researchers have explored novel multimodal probes (e.g., the fluorescence/MRI dual-modal probe) to achieve complementary parameters, making for a more accurate diagnosis and effective treatment of TBI [174,175]. Nonetheless, further research is needed to assess the potential applications of these imaging techniques in TBI diagnosis and management (Figure 2).

## 5. Challenges and Strategies Involved in Clinical Translation

The primary objective of preclinical or animal experimental TBI research is to deepen our understanding of TBI and its effects and to identify potential biomarkers for screening and therapies that can ultimately result in better-informed medical decisions and improved outcomes for TBI patients. However, one of the biggest challenges in TBI research, particularly mild TBI, is the clinical heterogeneity of injury patterns and severity. Choosing a model best suited for the research question is essential, since no single model can capture all possible features. Additionally, findings from one model may not be generalizable to other forms of TBI. One way to tackle this challenge is by adopting a second strategy, which involves incorporating multiple complementary models into the study design. This strategy is best implemented in a large-scale, pre-clinical, multi-institute consortium, which includes several injury models at various sites. A third strategy is to embrace the inherent variability of injuries caused by specific models of mild TBI and attempt to utilize injury variability by correlating the differences in animal responses to mild TBI with other outcomes of interest, such as biomarker expression or response to therapies [50].

Another critical consideration is incorporating clinically relevant or translatable outcomes into experiments, which is essential to improve the translation of TBI biomarkers and therapies. For example, methods such as diffusion MRI, MRS, fMRI, ASL, SWI, and PE currently used in clinical studies could also be applied to preclinical models [176,177,178]. Interestingly, some mild TBI preclinical neuroimaging evaluations, such as diffusion MRI and FDG-PET, have similarities to those reported in initial clinical mild TBI studies in preclinical research [176,177,179]. Finally, inter-species variability, such as differences in neurological deficits that persist for one week in rodents versus humans due to differences in life span, must be carefully characterized for compelling translational studies. Additionally, differences in the pathophysiological changes post-injury in the brain of both species must be considered [50]. In conclusion, effective clinical translation of TBI research requires addressing the challenges of clinical heterogeneity, incorporating clinically relevant outcomes, and carefully characterizing inter-species differences while utilizing complementary preclinical models and imaging techniques.

## 6. TBI in HIV Infection and Drug Abuse

Human immunodeficiency virus (HIV) infection has been associated with cognitive impairment and changes in cerebral metabolism. HIV can cause significant impairments in higher-order brain functions known as HIV-associated neurocognitive disorders (HAND), or NeuroHIV. While the causes of NeuroHIV persistence are unclear, certain comorbid factors may increase the brain’s vulnerability to HIV injury. Among these comorbidities, TBI is common [180]. TBI is often linked to acute alcohol intoxication, with reports suggesting that 30% to 50% of patients treated for TBI are usually intoxicated at the time of injury. Patients injured in motor vehicle accidents and assaults are more likely to be intoxicated [181]. The overall rate of alcohol use disorder (AUD) is high among patients who incur TBI, accounting for between one-third and half of all patients meeting the diagnostic criteria for AUD [182].

TBI has also been linked to drug abuse. Several studies have shown that individuals who have suffered a TBI are at an increased risk of developing a substance use disorder, particularly opioid use disorder. The relationship between TBI and drug abuse can be complex and bidirectional. For instance, individuals who abuse drugs or alcohol are likelier to experience a TBI due to their increased engagement in high-risk behaviors. Conversely, individuals who have suffered a TBI may develop a substance use disorder as a coping mechanism to manage the physical and emotional pain associated with the injury. Furthermore, research suggests that TBI may alter the brain’s reward system, making individuals more susceptible to drug addiction. TBI can also damage the brain’s prefrontal cortex and mesolimbic dopamine system, which play crucial roles in decision-making, impulse control, and reward processing. These changes may increase the risk of substance abuse and addiction following a TBI. TBI can also have significant and long-lasting effects on an individual’s brain function and increase the risk of developing substance use disorders, including alcohol and drug abuse.

## 7. Conclusions

TBI biomarkers can aid in early detection, predicting long-term outcomes, and early intervention to limit the progression of brain damage, as well as screening and avoiding unnecessary radiation exposure, making TBI a critical public health issue with significant economic implications. However, reliable and specific biomarkers for clinical use are currently limited and require extensive research. Despite the challenge, we cannot overstate the potential benefits of using TBI biomarkers for early detection, prediction, and intervention. We must continue our efforts to identify and validate potential biomarkers for clinical practice and improve diagnostic tools and understanding of TBI pathophysiology. In addition to improving diagnostic tools and understanding TBI pathophysiology, TBI biomarkers have several other future perspectives that could make an impact. One perspective is the potential use of TBI biomarkers in personalized medicine, where specific biomarkers could be associated with different TBI severities and outcomes, leading to more effective interventions and better patient outcomes. Another perspective is using TBI biomarkers in clinical trials for new treatments, enabling researchers to assess the efficacy of new treatments and develop more targeted therapies more accurately. Finally, developing TBI biomarkers could lead to advancements in sports medicine and concussion management by allowing for better assessment of injuries and more informed decisions about when athletes can safely return to play. Overall, the future perspectives for TBI biomarkers are promising, and continued research in this area could significantly impact clinical management and patient outcomes.

## Data Availability

Not applicable.
